# Virtual reality for reducing pain, anxiety, and distress in children, adolescents, and young adults with cancer: a systematic review and meta-analysis of randomized controlled trials

**DOI:** 10.3389/fpsyt.2026.1852113

**Published:** 2026-08-07

**Authors:** Shuai Yi, Yujiang Fan, Jie Ren, Zhaobing Zheng, Tingting Wang, Jiajia Bai

**Affiliations:** 1 Department of Radiation Oncology, The People’s Hospital of Huantai, Zibo, Shandong, China; 2 Department of Psychiatry, The People’s Hospital of Huantai, Zibo, Shandong, China; 3 Department of Pediatrics, The People’s Hospital of Huantai, Zibo, Shandong, China

**Keywords:** anxiety, distraction, meta-analysis, pain, pediatric oncology, virtual reality

## Abstract

**Background:**

Needle-related procedures are a significant source of pain, anxiety, and distress for children with cancer. This systematic review and meta-analysis aimed to evaluate the effectiveness of virtual reality (VR) as a distraction intervention for reducing these outcomes in pediatric oncology patients undergoing needle-related procedures.

**Methods:**

A systematic search of PubMed, Embase, Cochrane Central Register of Controlled Trials, PsycINFO, and CINAHL was conducted from database inception to March 23, 2026. We included randomized controlled trials (RCTs) evaluating the effect of VR (immersive or non-immersive) compared to standard care or active distraction in pediatric oncology patients (aged ≤21 years) undergoing needle-related procedures. The primary outcome was self-reported pain. Secondary outcomes included parent-reported pain, nurse-reported pain, self-reported fear, parent-reported fear, nurse-reported fear, self-reported anxiety, parent-reported anxiety, distress, procedure duration, and heart rate. Meta-analyses were performed using random-effects models. Risk of bias was assessed using the Cochrane RoB 2.0 tool.

**Results:**

Ten RCTs enrolling 536 participants were included. VR significantly reduced self-reported pain (Standardized Mean Difference [SMD] = -0.99, 95% Confidence Interval [CI]: -1.37 to -0.61, p = 0.001, I² = 71.7%), parent-reported pain (SMD = -1.10, 95% CI: -1.66 to -0.54, p < 0.001, I² = 83.6%), and nurse-reported pain (SMD = -1.00, 95% CI: -1.92 to -0.08, p = 0.025, I² = 80.0%). Significant reductions were also observed in self-reported fear (Weighted Mean Difference [WMD] = -1.10, 95% CI: -1.50 to -0.70, p < 0.001, I² = 58.0%), parent-reported fear (WMD = -1.31, 95% CI: -1.58 to -1.03, p < 0.001, I² = 7.1%), nurse-reported fear (WMD = -1.15, 95% CI: -1.55 to -0.75, p < 0.001, I² = 0.0%), self-reported anxiety (SMD = -1.06, 95% CI: -1.63 to -0.49, p = 0.002, I² = 79.9%), parent-reported anxiety (SMD = -1.59, 95% CI: -2.24 to -0.95, p < 0.001, I² = 51.5%), and distress (SMD = -0.35, 95% CI: -0.67 to -0.03, p = 0.032, I² = 19.6%). VR also significantly shortened procedure duration (WMD = -0.76 minutes, 95% CI: -1.34 to -0.19, p = 0.009, I² = 0.0%) but had no significant effect on heart rate (WMD = 1.57, 95% CI: -3.51 to 6.64, p = 0.544, I² = 80.3%). The overall risk of bias was low risk for five studies, some concerns for four studies, and high risk for one study.

**Conclusion:**

The available evidence suggests that VR may reduce pain, fear, anxiety, and distress and may shorten procedure duration in pediatric oncology patients undergoing needle-related procedures. These findings should be interpreted cautiously because several outcomes were based on few small trials and substantial heterogeneity was present. Larger multicenter RCTs are needed before routine implementation can be recommended.

**Systematic review registration:**

https://www.crd.york.ac.uk/PROSPERO/, identifier CRD42026134853.

## 1 Introduction

Pediatric cancer remains a significant global health challenge, with approximately 400,000 children diagnosed annually worldwide (1). Although advances in treatment have led to survival rates exceeding 80% in high-income countries (2), the intensive treatment protocols expose children to numerous painful and distressing medical procedures (3). Among these, needle-related procedures such as venipuncture and port access are cited by children as the most distressing aspect of their cancer journey (4–6). Unmanaged procedural pain and anxiety can have lasting negative consequences, including heightened pain sensitivity, avoidance of medical care, and the development of needle phobia that persists into adulthood (7–9). Therefore, effective pain and anxiety management during these procedures is a critical component of pediatric oncology care.

Pharmacological approaches, including topical anesthetics and conscious sedation, are commonly used but may not fully alleviate distress and can be associated with adverse effects (10). Consequently, non-pharmacological interventions, particularly distraction techniques, have gained increasing attention (11). Common non-pharmacological methods include relaxation/breathing exercises, storytelling, music listening, cartoon watching, kaleidoscopes, and guided imagery. Virtual reality (VR) offers distinct advantages over these traditional distractions due to its full sensory immersion, ability to block visual and auditory cues from the clinical environment, interactivity, and sustained attentional capture – features that conventional methods often lack. Distraction works by diverting the child’s attention away from the noxious stimuli, thereby reducing the cognitive resources available to process pain (12). The effectiveness of distraction is dependent on the degree to which it captures the child’s attention (13).

Virtual reality (VR) represents an advanced form of distraction that immerses the user in a computer-generated, interactive environment, engaging multiple senses simultaneously (14). Unlike traditional distractions (e.g., music, cartoons, kaleidoscopes, guided imagery), VR can completely block the user’s view of the clinical setting, creating a powerful sense of presence and immersion that enhances its analgesic and anxiolytic effects (15, 16). Functional MRI studies have shown that VR reduces pain-related brain activity by modulating attentional processes (17, 18). The growing accessibility and affordability of VR technology have made it a viable intervention in clinical settings (19, 20).

The application of VR in pediatric oncology has been explored in a number of studies, with promising but sometimes inconsistent findings. Some studies have reported significant reductions in pain and anxiety during port access and venipuncture (21, 22), while others have found non-significant trends or mixed results (23, 24). Variability in study design, VR technology (immersive vs. non-immersive), procedure type, and outcome measures have contributed to this inconsistency. Several systematic reviews have examined VR for procedural pain in children (25, 26), but few have focused specifically on pediatric oncology patients undergoing needle-related procedures. Moreover, previous reviews have often included a mix of study designs and have not provided quantitative syntheses of key outcomes such as fear and distress, nor have they evaluated outcomes reported by multiple informants (child, parent, nurse). Given the increasing volume of high-quality randomized controlled trials (RCTs) in this area, an updated and comprehensive meta-analysis is warranted. Therefore, this systematic review and meta-analysis aimed to answer the following research questions (1): Does VR distraction reduce self-reported pain in children and adolescents with cancer undergoing needle-related procedures? (2) Does VR reduce fear, anxiety, and distress as reported by children, parents, and nurses? (3) Does VR shorten procedure duration or affect physiological parameters such as heart rate?

## 2 Methods

### 2.1 Study registration and protocol

This systematic review and meta-analysis was conducted in accordance with the Preferred Reporting Items for Systematic Reviews and Meta-Analyses (PRISMA) statement (27). The protocol was registered in PROSPERO (registration number: CRD42026134853).

### 2.2 Search strategy

A comprehensive search was performed in five electronic databases: PubMed (MEDLINE), Embase (Ovid), Cochrane Central Register of Controlled Trials (CENTRAL), PsycINFO (EBSCOhost), and CINAHL (EBSCOhost). The search covered all records available from database inception up to March 23, 2026 (no lower date limit). The search strategy combined terms related to virtual reality (e.g., “virtual reality”, “VR”, “head-mounted display”), neoplasms (e.g., “cancer”, “tumor”, “leukemia”), pediatrics (e.g., “child”, “adolescent”, “pediatric”), pain and anxiety (e.g., “pain”, “anxiety”, “distress”), and RCTs (e.g., “randomized controlled trial”, “RCT”). The search was limited to English language publications. Detailed search strategies for each database are provided in **Table 1**.

**Table 1:** Full electronic search strategy.

1. PubMed (MEDLINE)	
Item	Details
Platform	PubMed (National Library of Medicine)
Search Period	Database inception to March 23, 2026
Language Restriction	English
Study Type Restriction	Randomized Controlled Trial
Search Strategy	#1 "Virtual Reality"[Mesh] #2 "virtual reality"[tiab] OR "VR"[tiab] OR "head-mounted display"[tiab] OR "immersive"[tiab] OR "computer-generated"[tiab] OR "simulation"[tiab] #3 #1 OR #2 #4 "Neoplasms"[Mesh] #5 "cancer"[tiab] OR "tumor"[tiab] OR "malignancy"[tiab] OR "leukemia"[tiab] OR "lymphoma"[tiab] OR "oncology"[tiab] #6 #4 OR #5 #7 "Child"[Mesh] #8 "child"[tiab] OR "children"[tiab] OR "adolescent"[Mesh] OR "adolescent"[tiab] OR "pediatric"[tiab] OR "paediatric"[tiab] OR "youth"[tiab] #9 #7 OR #8 #10 "Pain"[Mesh] #11 "pain"[tiab] OR "anxiety"[Mesh] OR "anxiety"[tiab] OR "distress"[tiab] OR "fear"[tiab] #12 #10 OR #11 #13 "Randomized Controlled Trial"[Publication Type] OR "controlled clinical trial"[pt] OR "randomized"[tiab] OR "placebo"[tiab] OR "RCT"[tiab] #14 #3 AND #6 AND #9 AND #12 AND #13 #15 #14 AND (english[Filter])
Number of Results	287

2. Embase (Ovid)	
Item	Details
Platform	Embase (Ovid)
Search Period	Database inception to March 23, 2026
Language Restriction	English
Study Type Restriction	Randomized Controlled Trial
Search Strategy	#1 'virtual reality'/exp #2 'virtual reality':ab,ti OR 'vr':ab,ti OR 'head mounted display':ab,ti OR 'immersive':ab,ti OR 'computer generated':ab,ti OR 'simulation':ab,ti #3 #1 OR #2 #4 'neoplasm'/exp #5 'cancer':ab,ti OR 'tumor':ab,ti OR 'malignancy':ab,ti OR 'leukemia':ab,ti OR 'lymphoma':ab,ti OR 'oncology':ab,ti #6 #4 OR #5 #7 'child'/exp #8 'child':ab,ti OR 'children':ab,ti OR 'adolescent'/exp OR 'adolescent':ab,ti OR 'pediatric':ab,ti OR 'paediatric':ab,ti OR 'youth':ab,ti #9 #7 OR #8 #10 'pain'/exp #11 'pain':ab,ti OR 'anxiety'/exp OR 'anxiety':ab,ti OR 'distress':ab,ti OR 'fear':ab,ti #12 #10 OR #11 #13 'randomized controlled trial'/exp OR 'controlled clinical trial':ab,ti OR 'randomized':ab,ti OR 'placebo':ab,ti OR 'rct':ab,ti #14 #3 AND #6 AND #9 AND #12 AND #13 #15 #14 AND [english]/lim
Number of Results	412

3. Cochrane Central Register of Controlled Trials (CENTRAL)	
Item	Details
Platform	Cochrane Library (Wiley)
Search Period	Database inception to March 23, 2026
Language Restriction	English
Study Type Restriction	Randomized Controlled Trial
Search Strategy	#1 (virtual reality OR VR OR head-mounted display OR immersive OR computer-generated OR simulation) #2 (cancer OR tumor OR malignancy OR leukemia OR lymphoma OR oncology) #3 (child OR children OR adolescent OR pediatric OR paediatric OR youth) #4 (pain OR anxiety OR distress OR fear) #5 #1 AND #2 AND #3 AND #4
Number of Results	156

4. PsycINFO (EBSCOhost)	
Item	Details
Platform	PsycINFO (EBSCOhost)
Search Period	Database inception to March 23, 2026
Language Restriction	English
Study Type Restriction	Randomized Controlled Trial
Search Strategy	S1 DE "Virtual Reality" S2 TI (virtual reality OR VR OR head-mounted display OR immersive OR computer-generated OR simulation) OR AB (virtual reality OR VR OR head-mounted display OR immersive OR computer-generated OR simulation) S3 S1 OR S2 S4 DE "Neoplasms" S5 TI (cancer OR tumor OR malignancy OR leukemia OR lymphoma OR oncology) OR AB (cancer OR tumor OR malignancy OR leukemia OR lymphoma OR oncology) S6 S4 OR S5 S7 DE "Childhood" OR DE "Adolescent Development" S8 TI (child OR children OR adolescent OR pediatric OR paediatric OR youth) OR AB (child OR children OR adolescent OR pediatric OR paediatric OR youth) S9 S7 OR S8 S10 DE "Pain" OR DE "Anxiety" S11 TI (pain OR anxiety OR distress OR fear) OR AB (pain OR anxiety OR distress OR fear) S12 S10 OR S11 S13 DE "Treatment Outcomes" OR DE "Randomized Controlled Trials" S14 TI (randomized OR RCT OR placebo) OR AB (randomized OR RCT OR placebo) S15 S13 OR S14 S16 S3 AND S6 AND S9 AND S12 AND S15 S17 S16 AND (LA "english")
Number of Results	94

5. CINAHL (EBSCOhost)	
Item	Details
Platform	CINAHL (EBSCOhost)
Search Period	Database inception to March 23, 2026
Language Restriction	English
Study Type Restriction	Randomized Controlled Trial
Search Strategy	S1 MH "Virtual Reality" S2 TI (virtual reality OR VR OR head-mounted display OR immersive OR computer-generated OR simulation) OR AB (virtual reality OR VR OR head-mounted display OR immersive OR computer-generated OR simulation) S3 S1 OR S2 S4 MH "Neoplasms+" S5 TI (cancer OR tumor OR malignancy OR leukemia OR lymphoma OR oncology) OR AB (cancer OR tumor OR malignancy OR leukemia OR lymphoma OR oncology) S6 S4 OR S5 S7 MH "Child+" OR MH "Adolescence+" S8 TI (child OR children OR adolescent OR pediatric OR paediatric OR youth) OR AB (child OR children OR adolescent OR pediatric OR paediatric OR youth) S9 S7 OR S8 S10 MH "Pain+" OR MH "Anxiety+" S11 TI (pain OR anxiety OR distress OR fear) OR AB (pain OR anxiety OR distress OR fear) S12 S10 OR S11 S13 MH "Randomized Controlled Trials+" S14 TI (randomized OR RCT OR placebo) OR AB (randomized OR RCT OR placebo) S15 S13 OR S14 S16 S3 AND S6 AND S9 AND S12 AND S15 S17 S16 AND (LA "english")
Number of Results	118

### 2.3 Inclusion and exclusion criteria

Studies were included if they met the following criteria: (1) study design: randomized controlled trials (including parallel, crossover, and within-subjects designs); (2) population: children, adolescents, and young adults (aged ≤21 years) with a diagnosis of cancer or onco-hematological disease (the upper age limit of 21 years follows common pediatric oncology practice, where young adults up to 21 years are often treated in pediatric oncology settings); (3) intervention: virtual reality (immersive or non-immersive) used as a distraction during a needle-related procedure (e.g., venipuncture, port access, lumbar puncture); (4) comparator: standard care, no intervention, or active distraction; (5) outcomes: at least one outcome related to pain, anxiety, fear, or distress; and (6) language: English. Studies were excluded if they were non-randomized, quasi-experimental, case series, or if they reported insufficient data for meta-analysis.

### 2.4 Data screening

Two reviewers independently screened the titles and abstracts of all retrieved records against the eligibility criteria using Covidence software. Disagreements were resolved through discussion or by consultation with a third reviewer. Full-text reports of potentially eligible studies were then independently assessed by the same two reviewers. Reasons for exclusion at the full-text stage were recorded and are presented in the PRISMA flow diagram.

### 2.5 Study selection and data extraction

Two reviewers independently performed all screening, data extraction, and risk of bias assessments. Any disagreements were resolved through consensus or consultation with a third reviewer. For the screening process, Covidence software was used for title/abstract screening and full-text review, and EndNote was used to remove duplicate records. Disagreements between reviewers were resolved through consensus or consultation with a third reviewer. Data extraction was performed independently by the same two reviewers using a pre-piloted Excel form. Extracted data included first author, year, country, study design, sample size, age, cancer type, procedure, VR type and content, outcomes, measurement tools, and main results. For continuous outcomes, means, standard deviations (SDs), and sample sizes were extracted. For studies reporting median and range, mean and SD were estimated using the method of Hozo et al. (28).

### 2.6 Risk of bias assessment

The risk of bias for each included study was assessed independently by two reviewers using the Cochrane RoB 2.0 tool (29). The tool evaluates five domains: (1) randomization process; (2) deviations from intended interventions; (3) missing outcome data; (4) outcome measurement; and (5) selective reporting. Each domain was rated as “low risk”, “some concerns” or “high risk”, and an overall risk of bias judgment was assigned. Disagreements were resolved through discussion.

### 2.7 Statistical analysis

Meta-analyses were performed using a random-effects model (DerSimonian-Laird) to account for expected heterogeneity between studies. For continuous outcomes, standardized mean differences (SMD) with 95% confidence intervals (CIs) were calculated when different scales were used, or weighted mean differences (WMD) when the same scale was used across studies. Heterogeneity was assessed using the I² statistic, with values of 25%, 50%, and 75% representing low, moderate, and high heterogeneity, respectively. Formal subgroup analyses to explore sources of heterogeneity (e.g., by VR type, age group, or control condition) were not performed because of the limited number of studies available. With only 10 RCTs included and nine of them using immersive VR, there were insufficient studies in any comparison category to yield meaningful statistical results. The random-effects model was therefore used to account for expected heterogeneity, and observed heterogeneity is described using I² statistics. Exploratory qualitative observations are provided in the Discussion. Sensitivity analyses were conducted by excluding studies with high risk of bias to assess the robustness of the findings. Additionally, to evaluate whether the inclusion of one study that used an active distraction comparator (iPad) instead of standard care influenced the results, we performed sensitivity analyses by excluding that study (23) from the meta-analyses for all relevant outcomes. Publication bias was assessed using funnel plots and Egger’s regression test when at least 8 studies were available. All statistical analyses were performed using Stata version 18.0.

## 3 Results

### 3.1 Study selection

**Figure 1:**
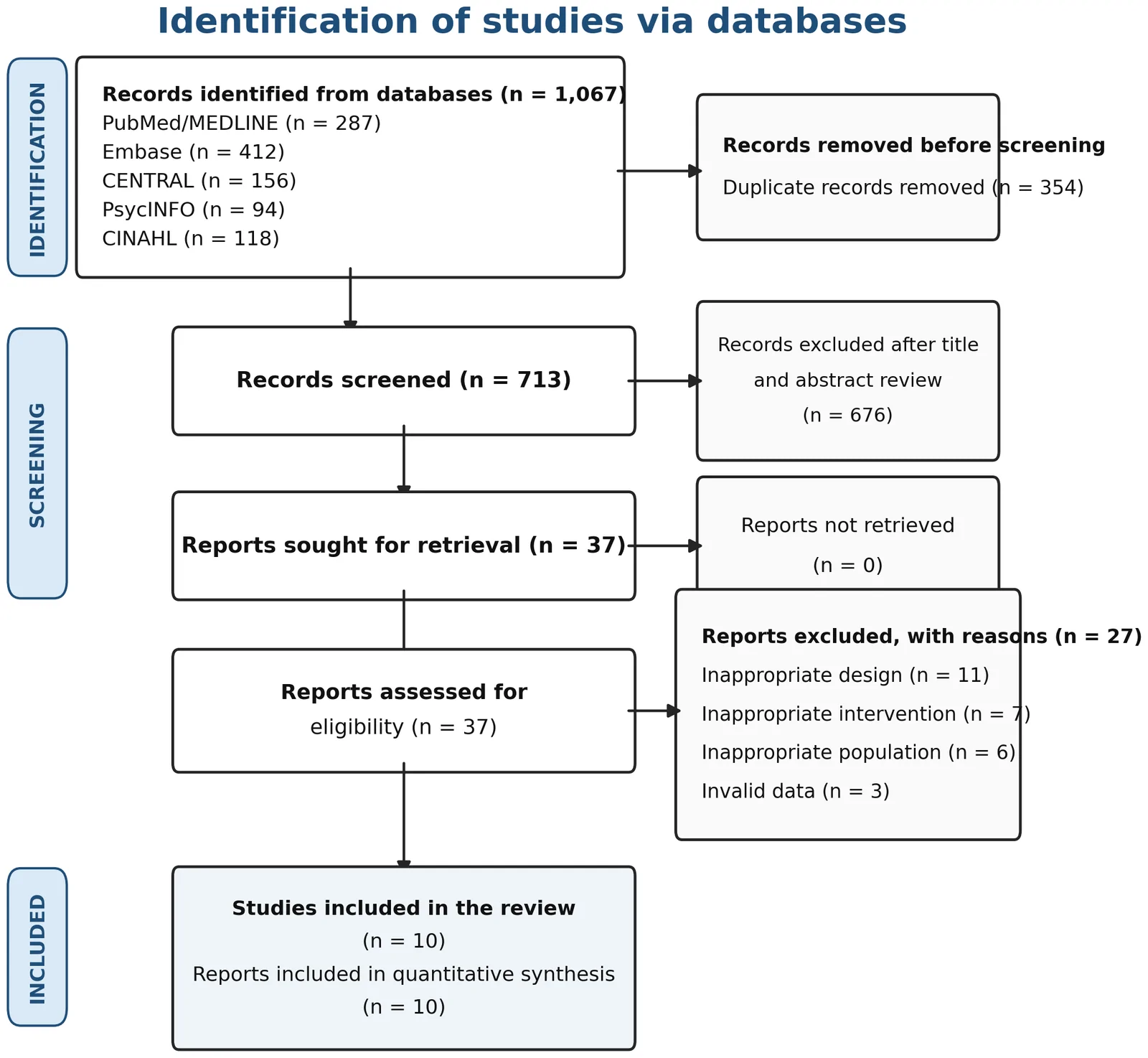
Flow chart of the literature search.

The systematic search yielded 1,067 records from the five databases. After removing 354 duplicates, 713 records were screened by title and abstract. Of these, 676 were excluded, leaving 37 full-text reports for eligibility assessment. Following full-text review, 27 reports were excluded: 11 for inappropriate study design, 7 for inappropriate intervention, 6 for inappropriate population, and 3 for invalid data. Ultimately, 10 studies (30–35) were included in the quantitative synthesis. The PRISMA flow diagram is shown in **Figure 1**.

### 3.2 Study characteristics

The 10 included RCTs were published between 2002 and 2024 and were conducted in Italy, Turkey, Canada, Sweden, Germany, the USA, and Hong Kong. Sample sizes ranged from 15 to 108 participants, with a total of 740 pediatric oncology patients. Ages ranged from 5 to 19 years. The most common needle-related procedures were port access (n=5 studies) and venipuncture (n=3 studies), with one study each on lumbar puncture and peripheral intravenous cannulation. VR interventions were predominantly immersive (n=9 studies), with one study using non-immersive VR. VR content varied, immersive VR using head-mounted displays with interactive games (e.g., SnowWorld, BioVirtualPed) or passive videos (rollercoaster, nature, cartoon); and non-immersive VR using a 3D display with a joystick-controlled game. Control conditions were primarily standard care without distraction, with one study using an iPad as an active comparator. Outcome measures included self-reported pain (n=8 studies), parent-reported pain (n=6), nurse-reported pain (n=2), fear (n=4), anxiety (n=4), distress (n=3), procedure duration (n=2), and heart rate (n=2). Detailed baseline characteristics and main results are presented in **Table 2**.

**Table 2:** **Characteristics of included trials**.

Author, year, country	Design	Sample size (VR/control)	Age, years (VR/control)	Cancer type	Procedure	VR type	VR content	Outcome measure(s)
Atzori et al., 2018, Italy (30)	Within-subject RCT	15 (within-subject)	10.92 ± 2.64	Onco-hematological disease	Venipuncture	Immersive	SnowWorld (interactive)	Pain (VAS)
Gerçeker et al., 2021, Turkey (21)	Parallel RCT	21/21	11.2 ± 3.1 / 11.7 ± 3.2	Onco-hematological disease	Port access	Immersive	Ocean Rift / Rollercoaster	Pain (WBS), fear (CFS), anxiety (CAM-S)
Hundert et al., 2022, Canada (23)	Pilot RCT	20/20	12.4 ± 3.2	ALL, brain tumor, lymphoma	Port access	Immersive	Underwater game	Pain (NRS), distress (NRS), fear (CFS)
Kanad et al., 2024, Turkey (35)	Parallel RCT	34/35	8.2 ± 2.72 / 8.0 ± 2.73	Onco-hematological disease	Venipuncture	Immersive	Epic Roller Coasters	Pain (WBS), fear (CFS)
Nilsson et al., 2009, Sweden (24)	Parallel RCT	21/21	5-18	Onco-hematological disease	Venipuncture / port access	Non-immersive	Hunt for Diamonds (game)	Pain (CAS), distress (FAS), heart rate
Reitze et al., 2024, Germany (31)	Crossover RCT	57 randomized; 38 completed both observations	6-18	Leukemia, thalassemia	Port / peripheral IV / Broviac	Immersive	Passive videos	Pain (NRS), anxiety (mYPAS-SF), distress (BAADS)
Savaş et al., 2024, Turkey (36)	Parallel RCT	31/31	9.33 ± 2.08 / 9.74 ± 1.76	Pediatric oncology	Port access	Immersive	BioVirtualPed (breathing control)	Pain (WBS), fear (CFS), anxiety (CAS)
Semerci et al., 2021, Turkey (22)	Parallel RCT	35/36	11.69 ± 3.36 / 11.67 ± 3.55	Pediatric oncology	Port access	Immersive	Rollercoaster (3D video)	Pain (WBS)
Wint et al., 2002, USA (33)	Pilot RCT	17/13	10-19	Pediatric oncology	Lumbar puncture	Immersive	Escape (3D video)	Pain (VAS)
Wong et al., 2021, Hong Kong (34)	Parallel RCT	54/54	10.2 ± 3.5 / 10.5 ± 3.8	Pediatric oncology	Peripheral IV cannulation	Immersive	Minions cartoon	Pain (VAS), anxiety (CSAS-C), heart rate

ALL, acute lymphoblastic leukemia; BAADS, Behavioral Approach-Avoidance and Distress Scale; CAM-S, Children's Anxiety Meter-State; CAS, Color Analogue Scale; CFS, Child Fear Scale; CSAS-C, Chinese State Anxiety Scale for Children; FAS, Facial Affective Scale; IV, intravenous; mYPAS-SF, modified Yale Preoperative Anxiety Scale-Short Form; NRS, Numerical Rating Scale; RCT, randomized controlled trial; VAS, Visual Analogue Scale; VR, virtual reality; WBS, Wong-Baker FACES Pain Rating Scale.

The measurement tools used across the included studies varied by outcome. For pain, the Visual Analogue Scale (VAS), Wong-Baker FACES Pain Rating Scale (WBS), Numeric Rating Scale (NRS), and Color Analogue Scale (CAS) were used. For anxiety, the Children’s Anxiety Meter-State (CAM-S), modified Yale Preoperative Anxiety Scale-Short Form (mYPAS-SF), and Chinese State Anxiety Scale for Children (CSAS-C) were employed. Fear was assessed using the Child Fear Scale (CFS). Distress was measured with the Facial Affective Scale (FAS) and the Behavioral Approach-Avoidance and Distress Scale (BAADS). These instruments were administered to children (self-report) as well as to parents and nurses (proxy-report or observation) according to each study’s protocol.

### 3.3 Risk of bias assessment

**Figure 2:**
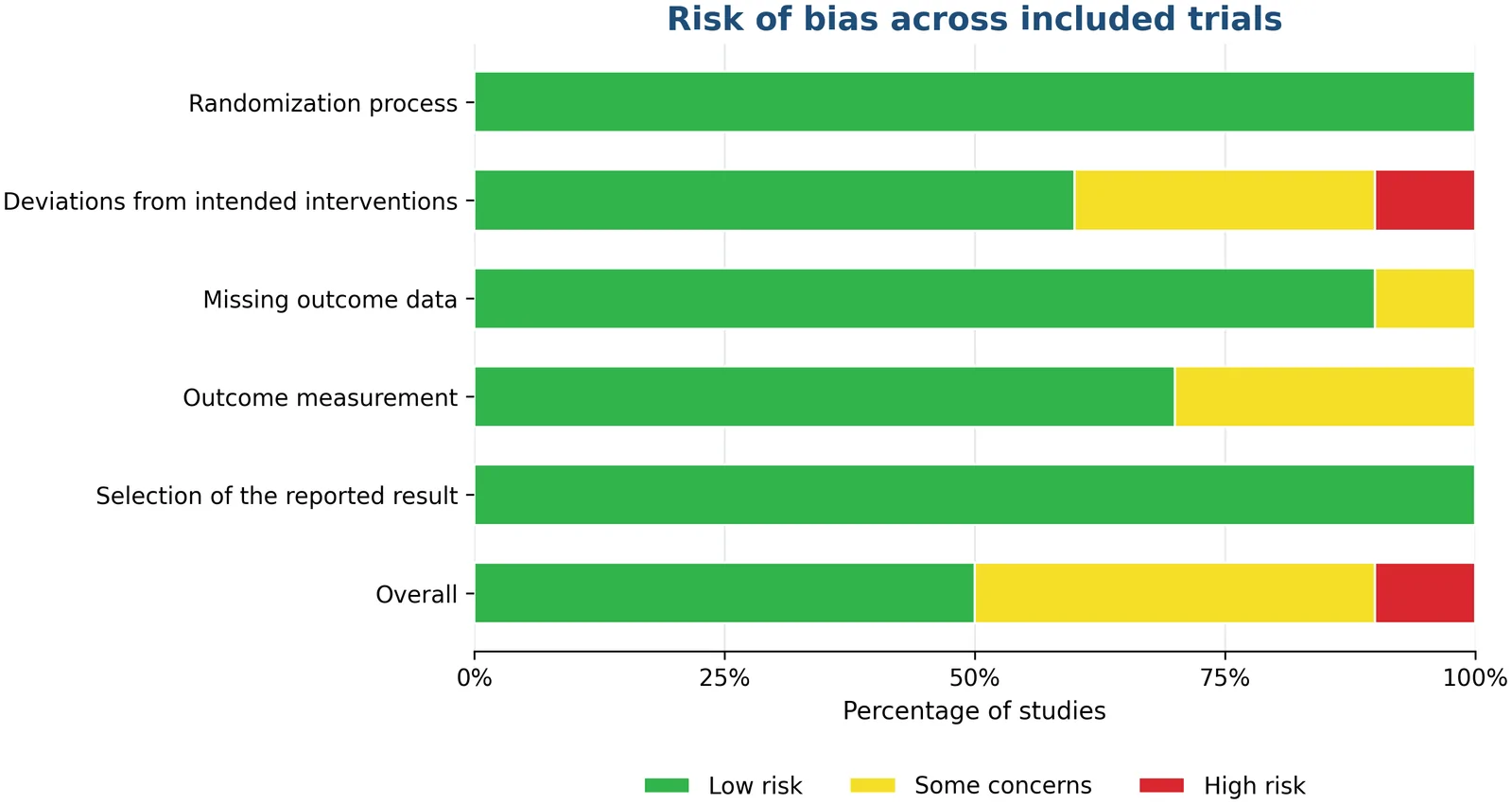
Risk of bias graph.

**Figure 3:**
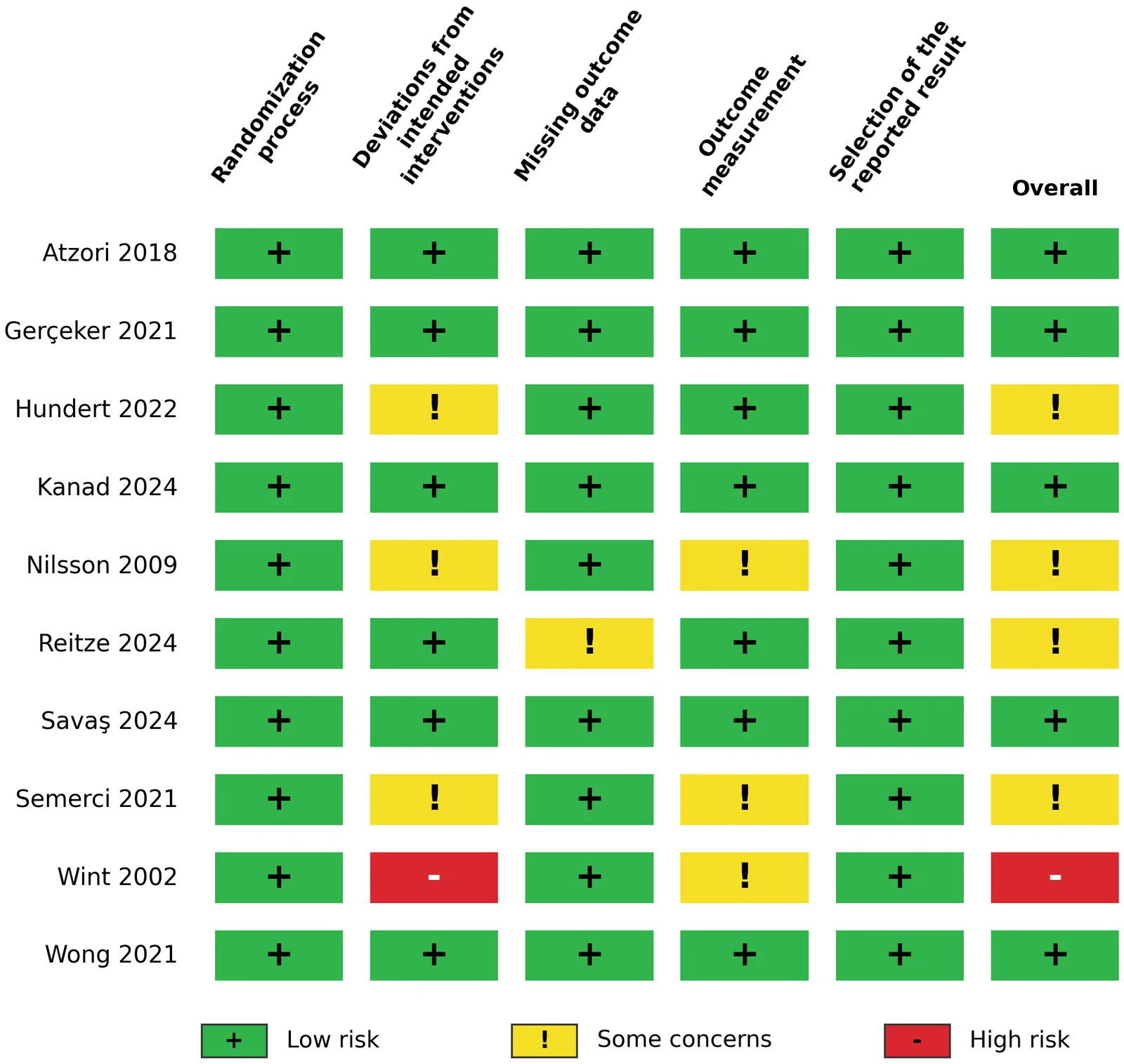
Risk of bias summary.

The overall risk of bias was low risk for five studies, some concerns for four studies, and high risk for one study (**Figures 2**, **3**). The high-risk study (33) was downgraded primarily due to lack of blinding and concerns about outcome measurement. The most common sources of “some concerns” were lack of blinding of participants and personnel (deviations from intended interventions) and unblinded outcome assessment (outcome measurement). All studies had low risk of bias for selective reporting, with most having registered protocols or reporting all pre-specified outcomes.

### 3.4 Effects of VR on pain

**Figure 4:**
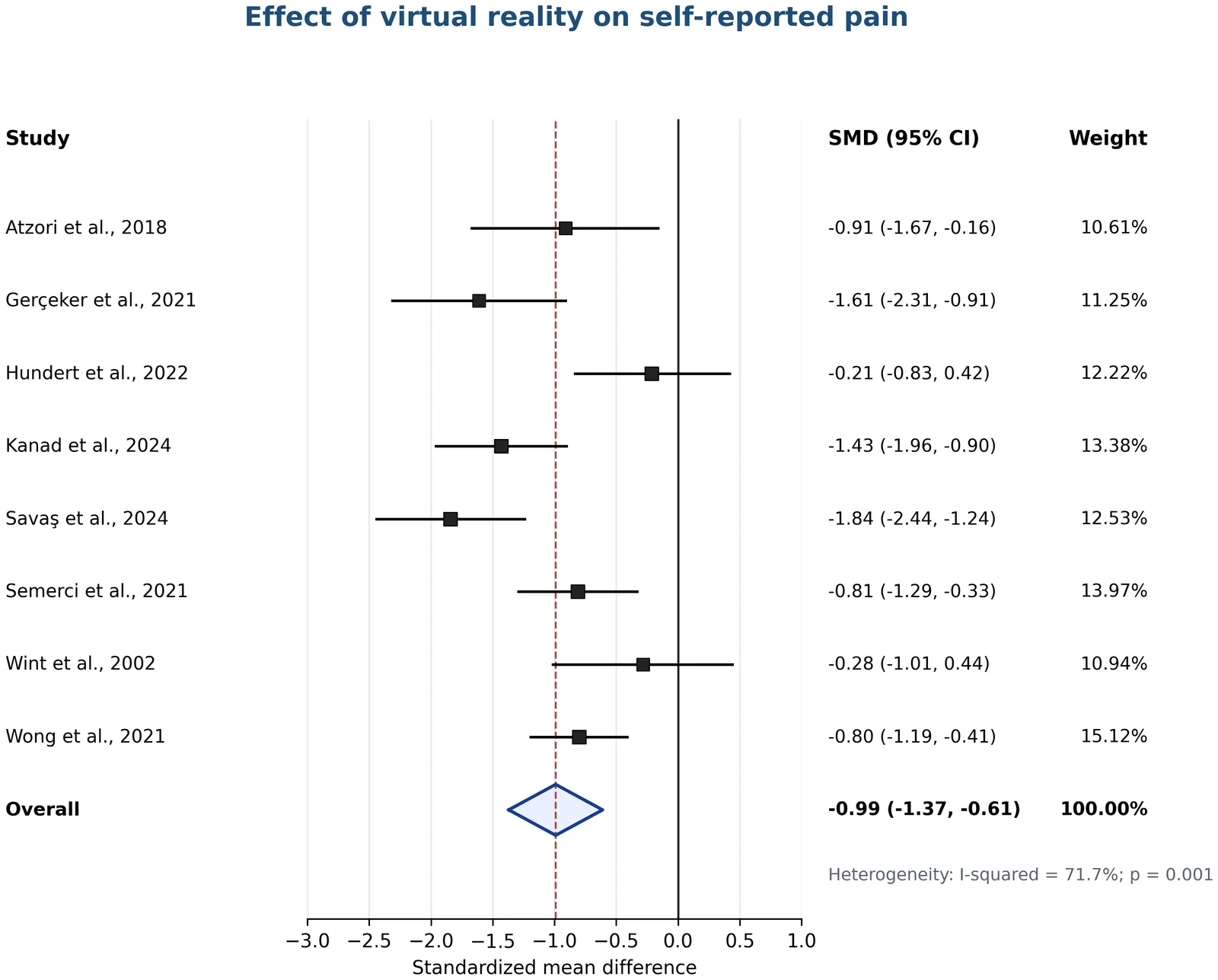
Forest plot of the effect of VR on self-reported pain.

For self-reported pain, eight studies contributed data. VR significantly reduced self-reported pain compared to control conditions (SMD = -0.99, 95% CI: -1.37 to -0.61, p = 0.001), with high heterogeneity (I² = 71.7%) (**Figure 4**). Sensitivity analyses excluding the high-risk study did not substantially alter the findings for self-reported pain (**Supplementary Figure 4**). Egger’s regression test for publication bias yielded a non-significant result (p = 0.872). Qualitatively, only one included study used non-immersive VR (Nilsson et al., 2009) (24), and it reported non-significant effects on self-reported pain. All other studies used immersive VR and showed significant pain reduction. This suggests that immersive VR may be a key driver of the overall effect, although this hypothesis requires confirmation in future studies with direct comparisons.

**Figure 5:**
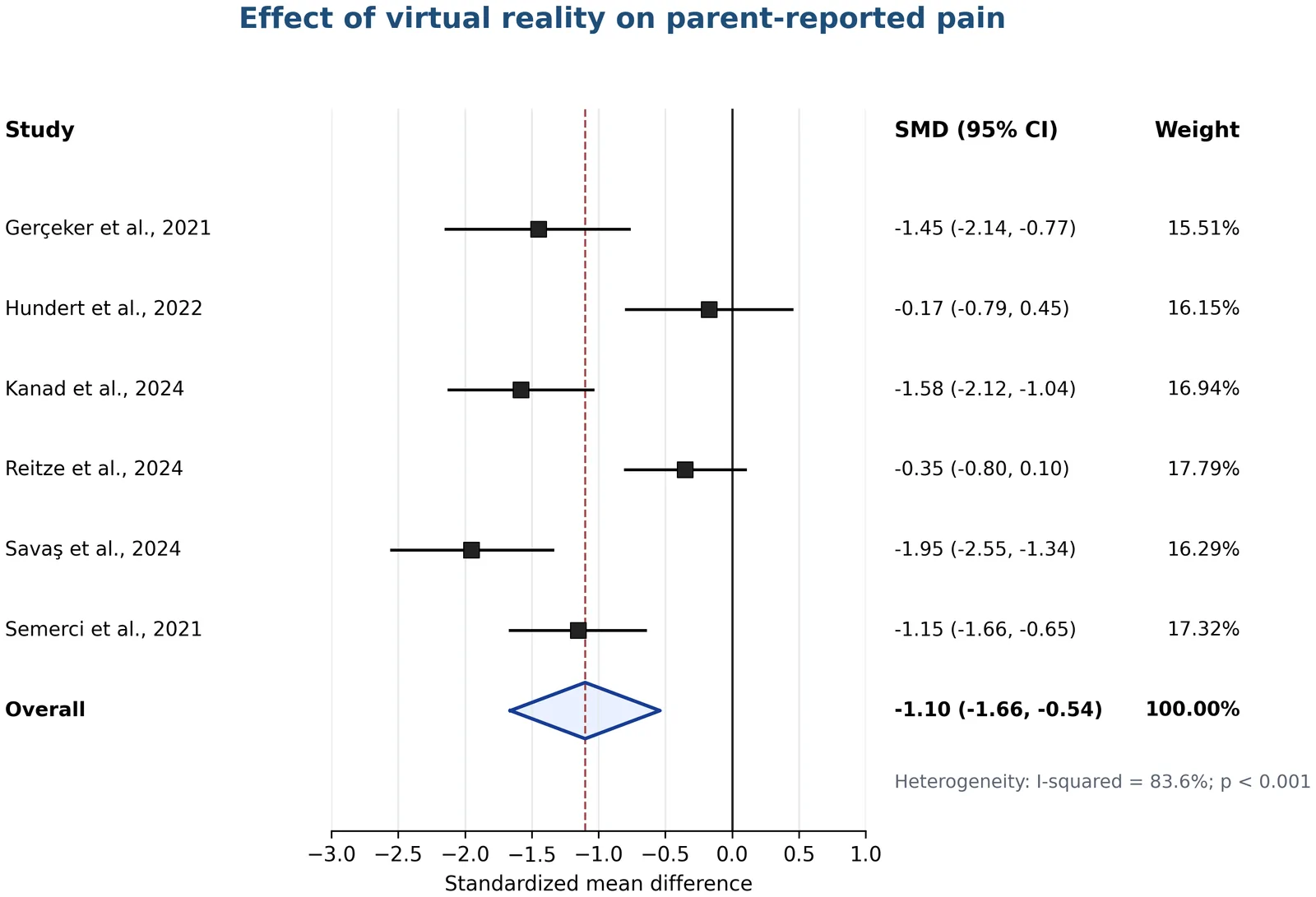
Forest plot of the effect of VR on parents-reported pain.

For parent-reported pain, six studies were included. VR significantly reduced parent-reported pain (SMD = -1.10, 95% CI: -1.66 to -0.54, p < 0.001), with substantial heterogeneity (I² = 83.6%) (**Figure 5**). Sensitivity analyses excluding the high-risk study did not substantially alter the findings for parent-reported pain (**Supplementary Figure 5**). Egger’s test showed no evidence of publication bias (p = 0.419).

**Figure 6:**
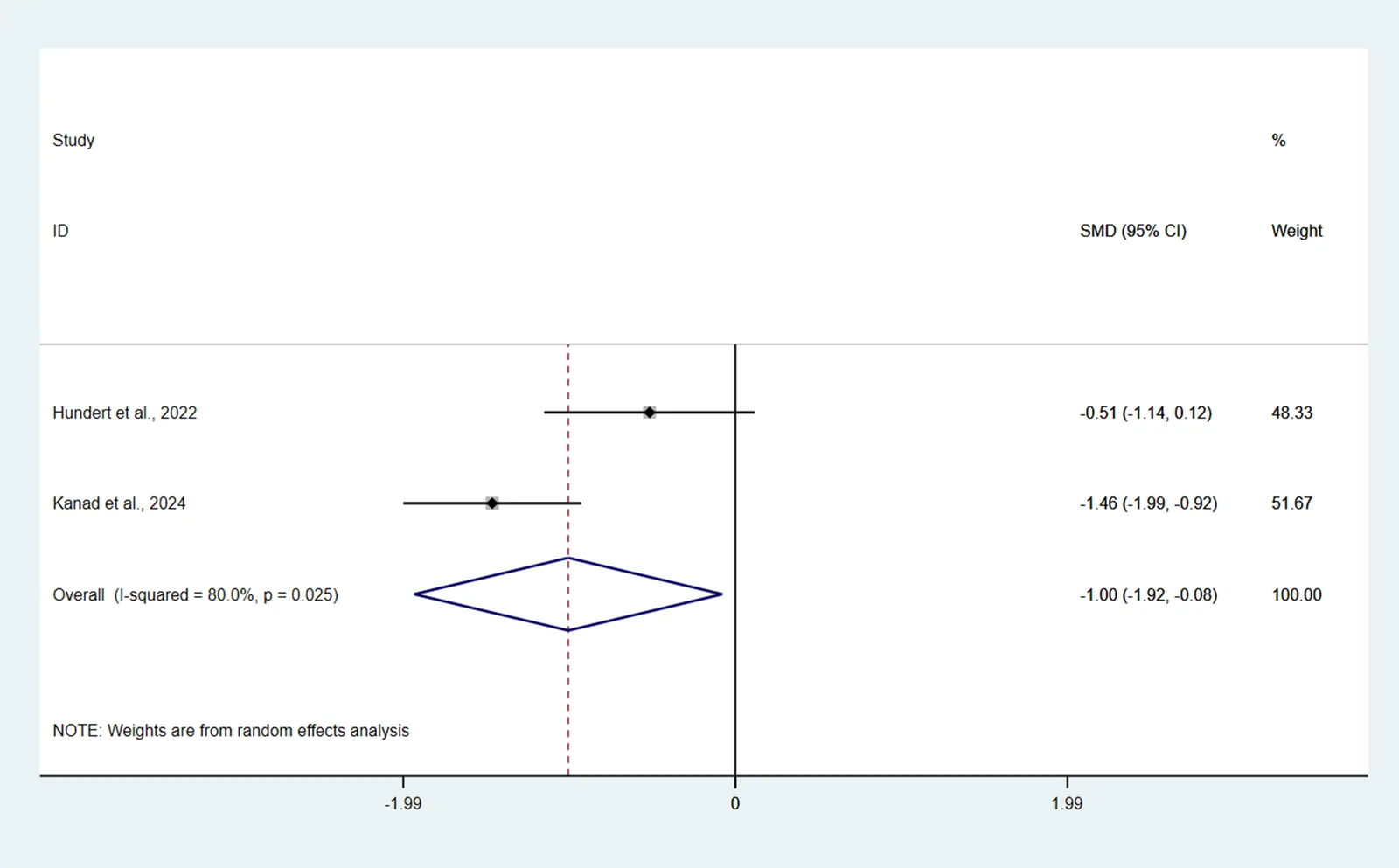
Forest plot of the effect of VR on nurse-reported pain.

For nurse-reported pain, only two studies provided data. VR significantly reduced nurse-reported pain (SMD = -1.00, 95% CI: -1.92 to -0.08, p = 0.033), with high heterogeneity (I² = 80.0%) (**Figure 6**). However, the evidence for nurse-reported pain is based on only two studies; therefore, this finding should be interpreted as preliminary and requires confirmation in larger trials.

### 3.5 Effects of VR on fear

**Figure 7:**
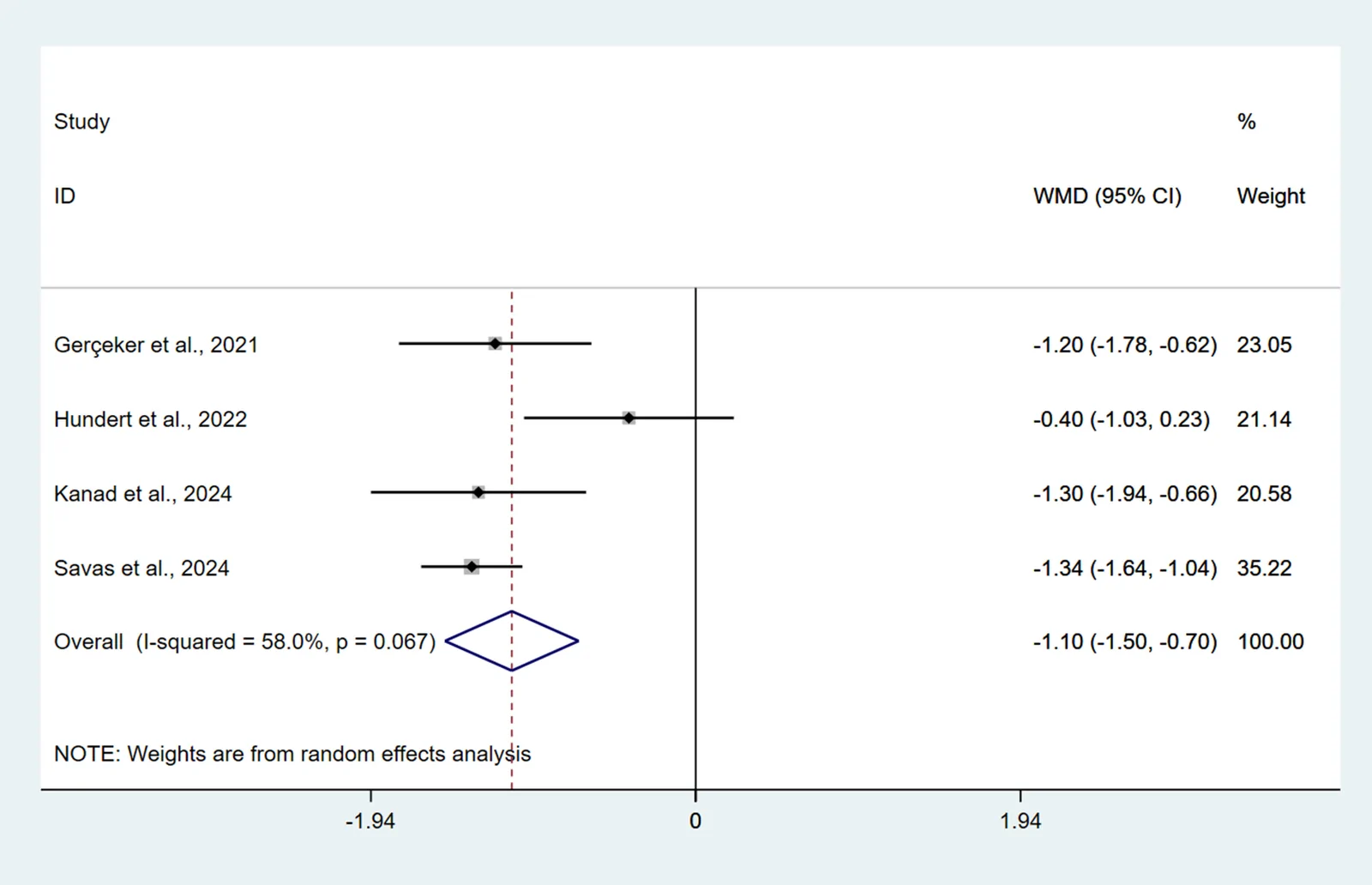
Forest plot of the effect of VR on self-reported fear.

For self-reported fear, four studies were analyzed. VR significantly reduced self-reported fear (WMD = -1.10, 95% CI: -1.50 to -0.70, p < 0.001), with moderate heterogeneity (I² = 58.0%) (**Figure 7**). Sensitivity analyses confirmed the robustness (**Supplementary Figure 6**). Egger’s regression test was non-significant (p = 0.714).

**Figure 8:**
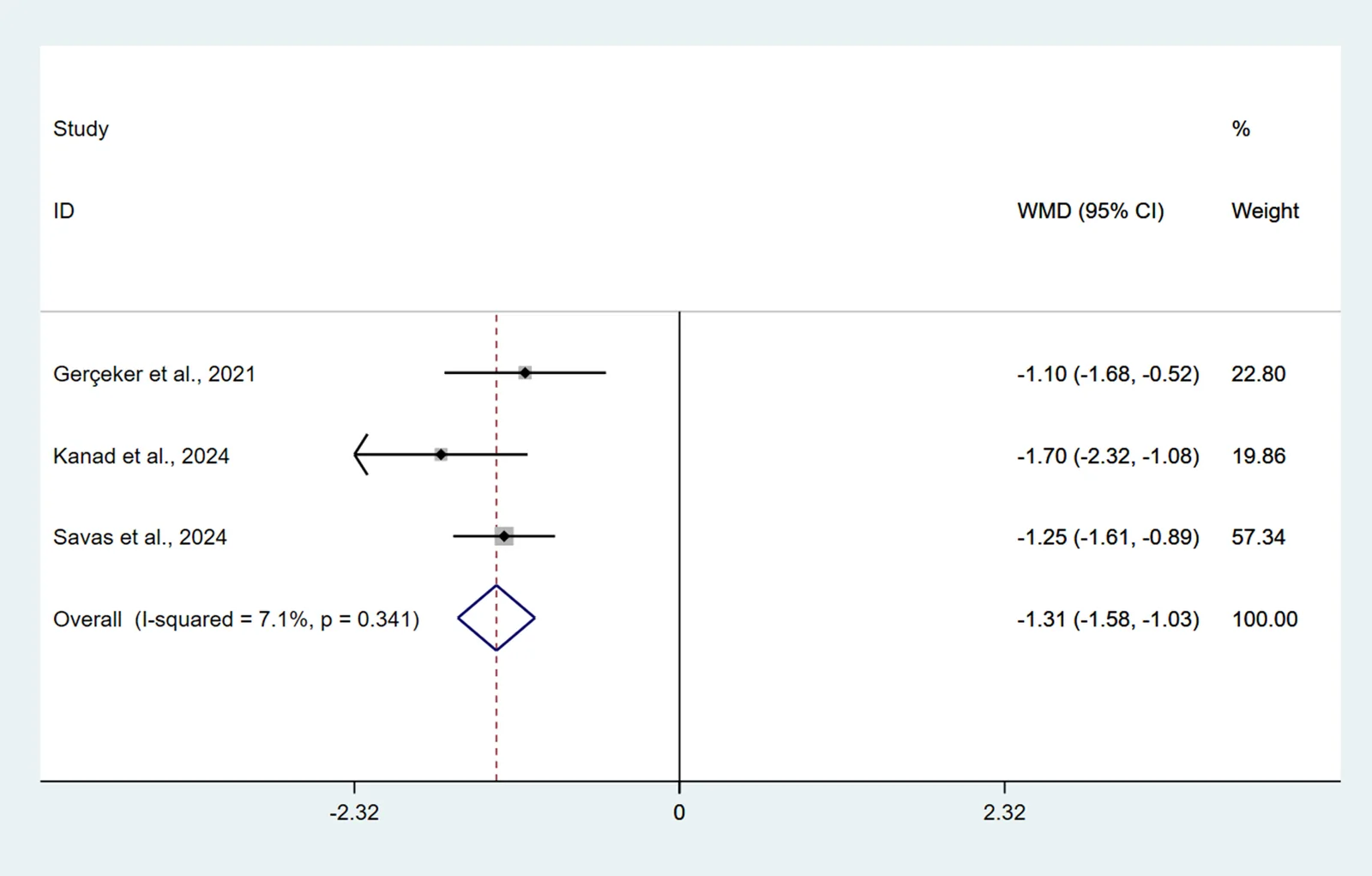
Forest plot of the effect of VR on parents-reported fear.

For parent-reported fear, three studies contributed. VR significantly reduced parent-reported fear (WMD = -1.31, 95% CI: -1.58 to -1.03, p < 0.001), with low heterogeneity (I² = 7.1%) (**Figure 8**). Sensitivity analyses confirmed the robustness (**Supplementary Figure 7**). Publication bias assessment was not performed due to the limited number of studies (<10).

**Figure 9:**
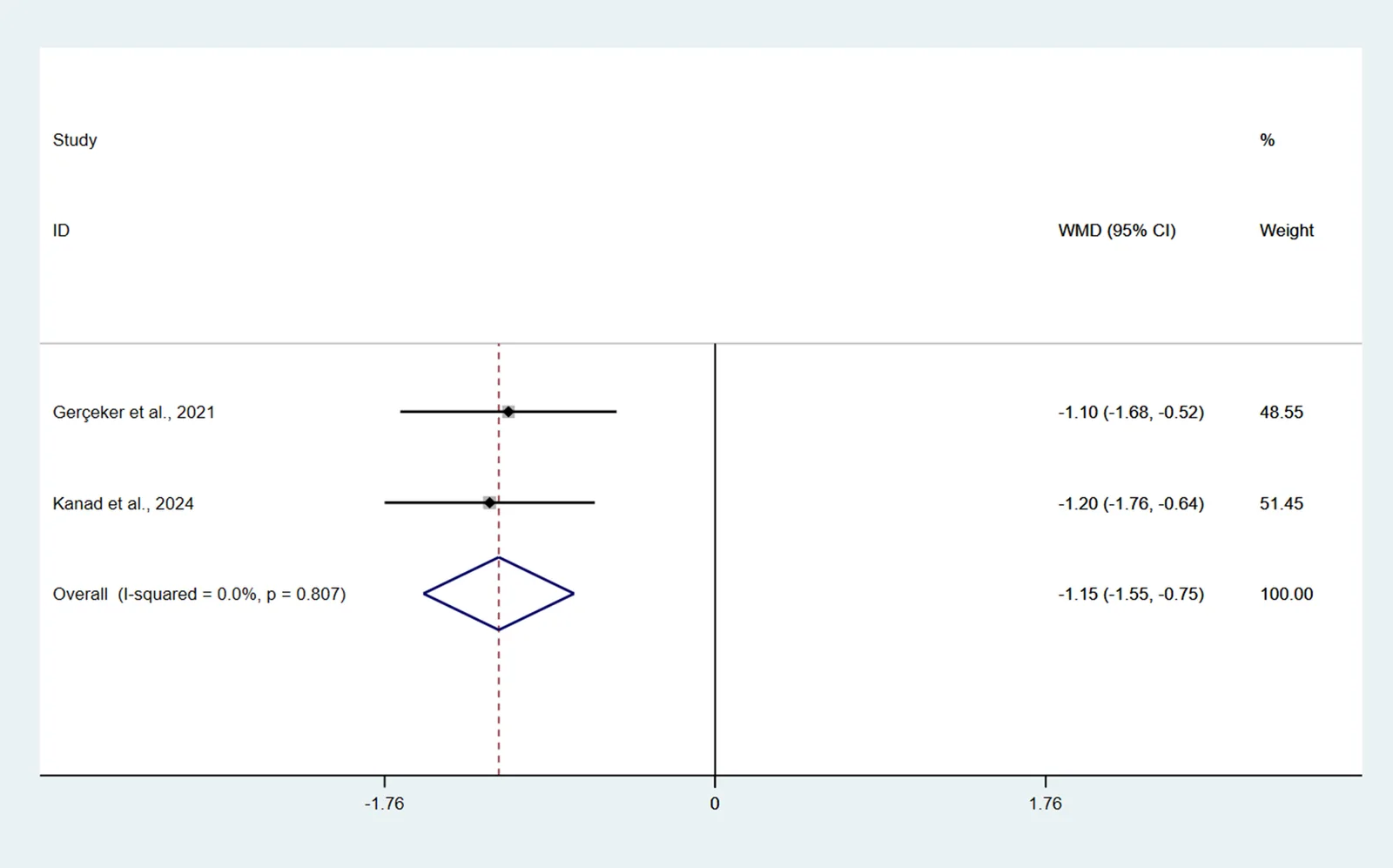
Forest plot of the effect of VR on nurse-reported fear.

For nurse-reported fear, two studies provided data. VR significantly reduced nurse-reported fear (WMD = -1.15, 95% CI: -1.55 to -0.75, p < 0.001), with no heterogeneity (I² = 0.0%) (**Figure 9**). Because only two studies were available, publication bias was not assessed.

### 3.6 Effects of VR on anxiety

**Figure 10:**
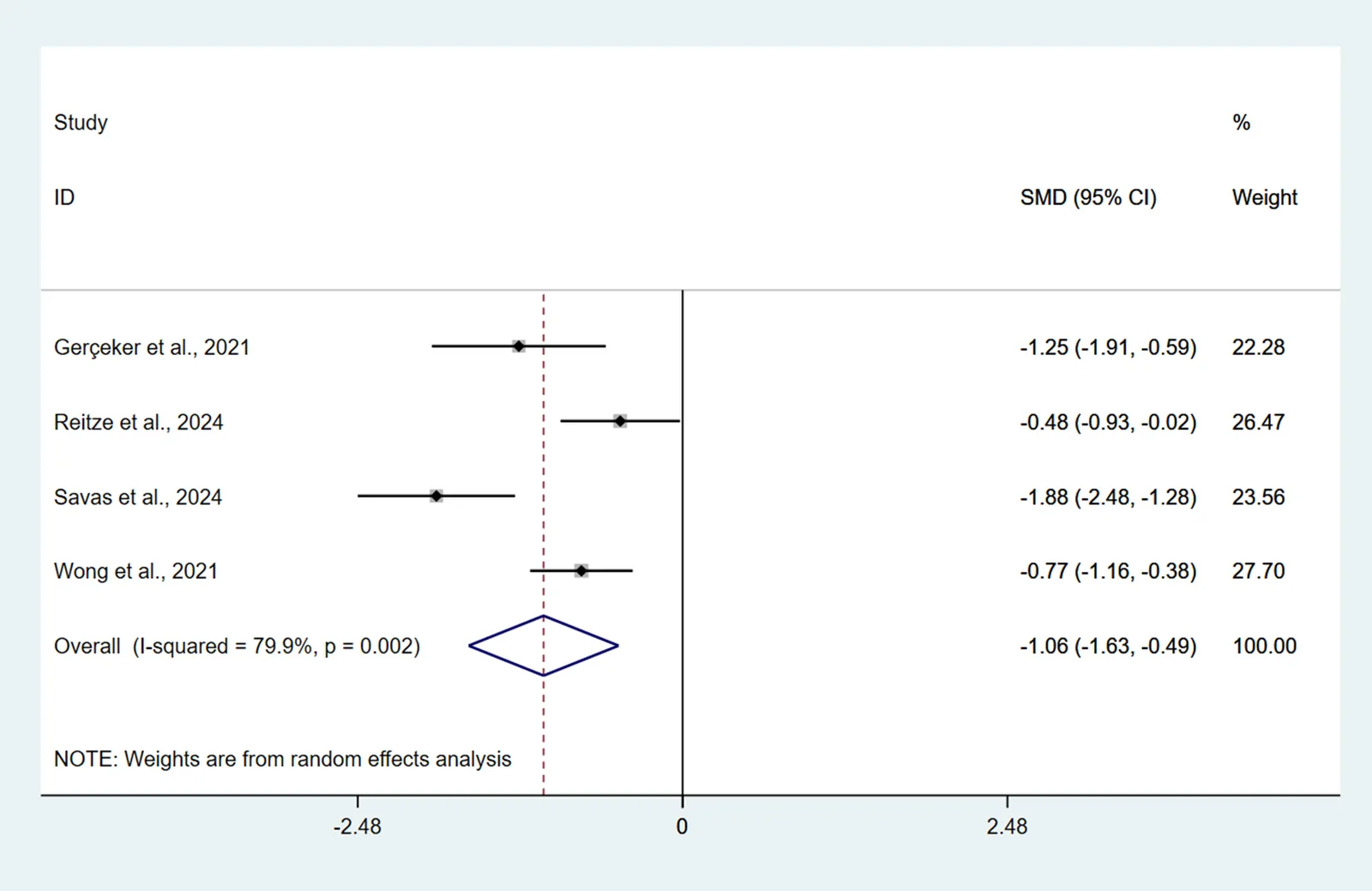
Forest plot of the effect of VR on self-reported anxiety.

For self-reported anxiety, four studies were included. VR significantly reduced self-reported anxiety (SMD = -1.06, 95% CI: -1.63 to -0.49, p = 0.002), with high heterogeneity (I² = 79.9%) (**Figure 10**). Sensitivity analyses indicated that the overall effect for self-reported anxiety remained significant when any single study was omitted (**Supplementary Figure 8**). Egger’s test was non-significant (p = 0.271).

**Figure 11:**
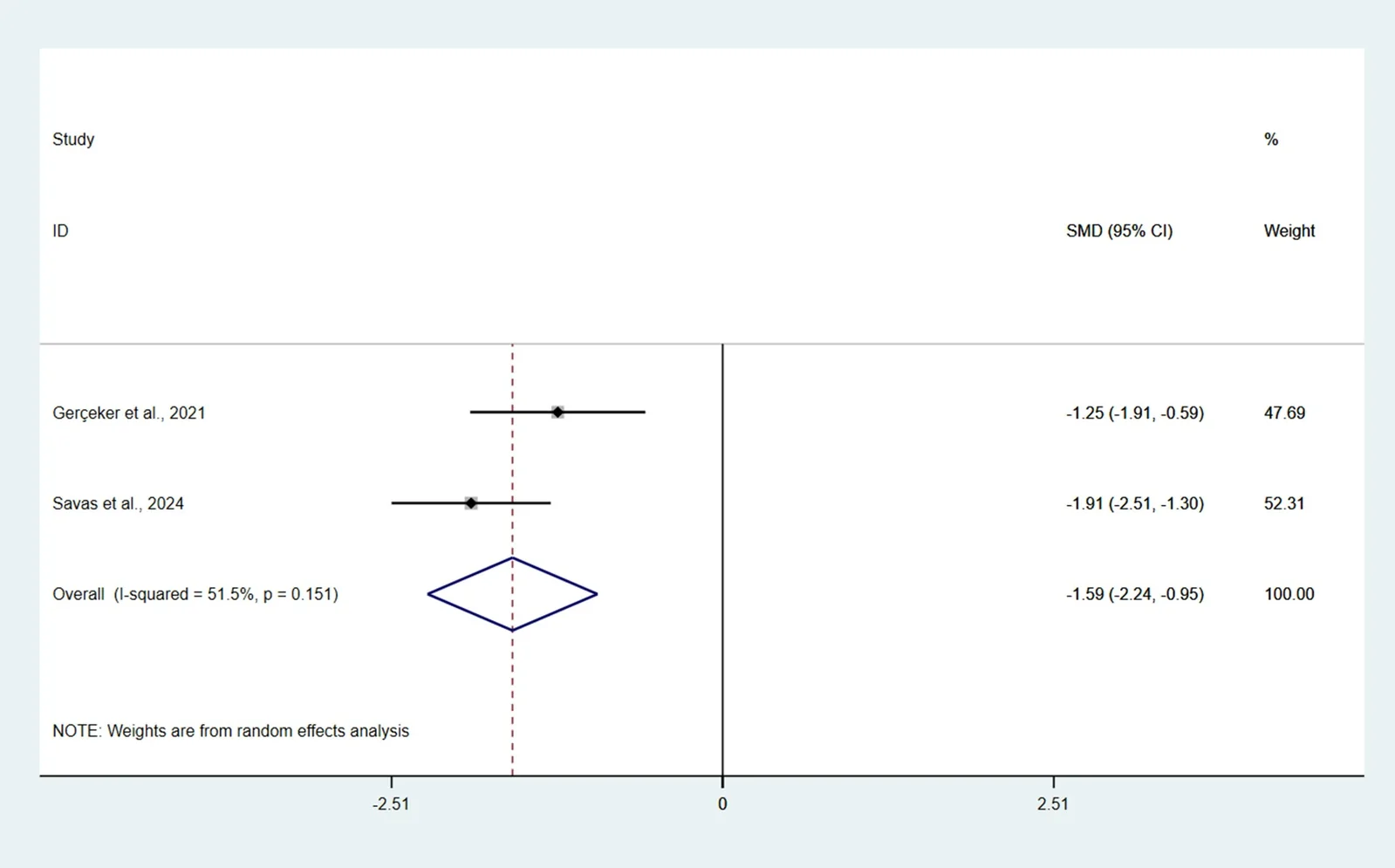
Forest plot of the effect of VR on parents-reported anxiety.

For parent-reported anxiety, two studies contributed data. VR significantly reduced parent-reported anxiety (SMD = -1.59, 95% CI: -2.24 to -0.95, p < 0.001), with moderate heterogeneity (I² = 51.5%) (**Figure 11**). Publication bias was not assessed due to the small number of studies.

### 3.7 Effects of VR on distress

For distress, three studies provided data. VR significantly reduced distress (SMD = -0.35, 95% CI: -0.67 to -0.03, p = 0.032), with low heterogeneity (I² = 19.6%) (**Supplementary Figure 1**). Sensitivity analyses suggested that the effect was consistent and not driven by any single study (**Supplementary Figure 9**). Egger’s test was non-significant (p = 0.896).

### 3.8 Effects of VR on procedure duration and heart rate

VR significantly shortened procedure duration (two studies, WMD = -0.76 minutes, 95% CI: -1.34 to -0.19, p = 0.009, I² = 0.0%) (**Supplementary Figure 2**). No significant effect was observed on heart rate (two studies, WMD = 1.57, 95% CI: -3.51 to 6.64, p = 0.544, I² = 80.3%) (**Supplementary Figure 3**). Given that only two studies contributed to this outcome, the lack of a significant effect does not constitute strong evidence of absence; further research is needed.

### 3.9 Publication bias

Egger’s regression test was performed for completeness, but its power is known to be low with fewer than 10 studies. Therefore, the non-significant results (self-reported pain p = 0.872, parent-reported pain p = 0.419, self-reported anxiety p = 0.271, self-reported fear p = 0.714, and distress p = 0.896) should not be interpreted as definitive evidence of absence of publication bias. Begg’s test results were also non-significant for all outcomes. Visual inspection of the funnel plot for self-reported pain (**Supplementary Figure 10**) showed no substantial asymmetry.

## 4 Discussion

This systematic review and meta-analysis of 10 RCTs comprising 740 pediatric oncology patients provides robust evidence that virtual reality is an effective distraction intervention for reducing pain, fear, anxiety, and distress during needle-related procedures. The magnitude of the effects was large for self-reported pain (SMD = -0.99), parent-reported pain (SMD = -1.10), and fear across all reporters (WMDs from -1.10 to -1.31), and moderate for anxiety (SMD = -1.06) and distress (SMD = -0.35). The consistent pattern of significant effects across multiple informants (child, parent, nurse) strengthens the validity of these findings and suggests that the benefits of VR are perceptible to all stakeholders involved in the procedure. The significant reduction in procedure duration (by approximately 0.76 minutes) is clinically meaningful, as shorter procedures may reduce overall stress and allow for more efficient clinical workflows. The lack of a significant effect on heart rate, despite significant reductions in self-reported anxiety and distress, may reflect the complex interplay between physiological and psychological responses; however, given that only two studies contributed to this outcome, this finding should be regarded as preliminary. Similarly, the significant reduction in nurse-reported pain is based on only two studies and requires cautious interpretation.

These findings have important implications for clinical practice in pediatric oncology. Before recommending routine clinical integration, safety considerations must be addressed. Across the included studies, adverse effects of VR were infrequent and generally mild. Most studies reported no VR-related side effects. Where adverse events were documented, transient dizziness or nausea occurred in fewer than 5% of participants, and no serious adverse events (e.g., severe cybersickness, falls, or prolonged symptoms) were reported. However, children undergoing active chemotherapy or those with pre-existing vestibular dysfunction, migraine, or seizure disorders may be more susceptible to VR-induced symptoms. Therefore, we recommend a brief pre-procedural screening for such conditions and limiting VR session duration to ≤10 minutes for younger or vulnerable children. With these precautions, VR remains a safe and well-tolerated non-pharmacological intervention that offers engaging, age-appropriate distraction easily implemented in outpatient clinics and inpatient settings (37, 38). Importantly, the high acceptability of VR reported in the included studies, with up to 95% of children expressing enjoyment and desire to use it again (31), suggests that it is well-received by young patients. The feasibility of implementation is supported by the successful use of commercially available VR headsets and software, which are increasingly affordable. For healthcare providers, VR can facilitate procedure completion by reducing patient movement and resistance, thereby improving success rates and reducing the need for multiple attempts or additional sedation (39). Clinicians should consider incorporating VR into pre-procedure preparation protocols and offering it as a first-line distraction option for children undergoing repeated needle procedures.

Our findings are consistent with and extend previous systematic reviews on VR for procedural pain in children. A Cochrane review by Lambert et al. (15) concluded that VR may be beneficial for acute pain in children, but noted that the quality of evidence was low due to heterogeneity and risk of bias. Our review, which focused specifically on pediatric oncology and included only RCTs, provides a higher level of evidence. Similarly, a meta-analysis by Eijlers et al. (25) reported that VR was effective for reducing pain and anxiety in children undergoing medical procedures, but their analysis included a mix of populations and procedures. Our findings are also in line with individual RCTs that have reported positive effects of VR on pain during port access (21, 40) and venipuncture (41, 42). However, our meta-analysis also identified studies with non-significant trends (23, 24), highlighting the importance of pooling data to achieve sufficient statistical power. The inclusion of outcomes such as fear and distress in our analysis adds novel insights, as these are often overlooked but are critical components of the procedural experience for children (43, 44).

Several limitations should be acknowledged. First, there was substantial heterogeneity across studies for several outcomes (I² > 70% for pain and anxiety), which may be attributable to differences in VR technology (immersive vs. non-immersive, interactive vs. passive), procedure type, patient age, and control conditions. While random-effects models account for this heterogeneity, the results should be interpreted with caution. Another limitation is the inability to perform formal subgroup analyses to explore the high statistical heterogeneity observed for pain and anxiety outcomes. With only 10 RCTs and nine using immersive VR, there were insufficient studies in each subgroup category to conduct meaningful meta-regression or subgroup comparisons. Therefore, while random-effects models were used to account for heterogeneity, the sources of variation (e.g., VR immersion level, patient age, procedure type) remain uncertain and should be investigated in future larger trials. Second, blinding of participants and personnel was not feasible in most studies due to the nature of the VR intervention, introducing potential performance bias. However, the use of observer-rated outcomes and the consistency of results across different reporters (including self-report) mitigate this concern to some extent. Third, the sample sizes of individual studies were generally small, with several studies having less than 50 participants per group. Fourth, While the included studies originated from diverse settings (Italy, Turkey, Canada, Sweden, Germany, USA, and Hong Kong), the majority were single-center studies, which may limit generalizability to broader populations and healthcare systems. Fifth, the lack of standardized outcome measures across studies necessitated the use of SMDs for some analyses, which are less clinically interpretable than mean differences. Sixth, the risk of bias assessment identified one study as high risk, and while sensitivity analyses showed that its exclusion did not alter the main findings, it remains a potential source of bias. Seventh, restricting the search to English-language publications may have introduced language bias, as relevant trials published in other languages could have been missed. Eighth, conclusions regarding nurse-reported pain and heart rate are drawn from a very limited number of studies (only two each), and thus should be considered hypothesis-generating rather than definitive. Larger prospective trials are needed to confirm these preliminary findings. Ninth, one included study (23) used an active distractor (iPad) rather than standard care as the comparator. Although this differs from the other included studies, our sensitivity analyses showed that excluding this study did not meaningfully alter the pooled effect sizes for any outcome, suggesting that its inclusion did not bias the overall estimates. Nevertheless, the heterogeneity in comparator types should be considered when interpreting the results. Tenth, the assessment of publication bias using Egger’s regression test was limited by the small number of studies (fewer than 10 for all outcomes), which reduces the statistical power to detect true asymmetry. Consequently, the non-significant results do not rule out the possibility of publication bias.

Future research should address the limitations of existing studies. Large, multi-center, adequately powered RCTs with standardized outcome measures are needed to confirm these findings and explore moderators of treatment effect, such as age, cancer type, procedure type, and VR characteristics (e.g., immersive vs. non-immersive, interactive vs. passive). The wide age range of participants (5-19 years) introduces developmental heterogeneity; younger children may have different cognitive capacities for engaging with VR and understanding distraction instructions. Future studies should therefore stratify analyses by age group (e.g., preschool, school-age, adolescent) to explore differential effects. Comparisons of VR with other active distraction methods (e.g., tablet games, music) would help determine whether VR offers incremental benefits over less expensive technologies. The optimal timing and duration of VR exposure also warrant investigation. Additionally, long-term outcomes, including the impact of VR on the development of needle phobia and treatment adherence, should be examined. Cost-effectiveness analyses are needed to support the economic case for implementing VR in routine care. Finally, as VR technology continues to evolve, studies should evaluate the effectiveness of newer, more immersive platforms and explore the potential of biofeedback-based VR (36), which may enhance engagement and outcomes by allowing children to regulate their physiological state through the virtual environment.

## 5 Conclusion

This systematic review and meta-analysis suggests that VR may reduce pain, fear, anxiety, and distress and may shorten procedure duration in pediatric oncology patients undergoing needle-related procedures. However, the findings should be interpreted cautiously because most outcomes were informed by small numbers of studies, several pooled estimates showed substantial heterogeneity, and certainty of evidence was not formally assessed. Larger multicenter RCTs using standardized outcome measures are needed before routine implementation can be recommended.

## Data Availability

The original contributions presented in the study are included in the article/**Supplementary Material**. Further inquiries can be directed to the corresponding authors.

## Supplementary material

Supplementary Figure 1 Forest plot of the effect of VR on distress.

Supplementary Figure 2 Forest plot of the effect of VR on duration.

Supplementary Figure 3 Forest plot of the effect of VR on heart rate.

Supplementary Figure 4 Sensitivity analysis of VR on self-report pain.

Supplementary Figure 5 Sensitivity analysis of VR on parents-report pain.

Supplementary Figure 6 Sensitivity analysis of VR on self-report fear.

Supplementary Figure 7 Sensitivity analysis of VR on parents-report fear.

Supplementary Figure 8 Sensitivity analysis of VR on self-report anxiety.

Supplementary Figure 9 Sensitivity analysis of VR on distress.

Supplementary Figure 10 Funnel plot of VR on self-report pain.
